# Huntington disease alters the actionable information in plasma extracellular vesicles

**DOI:** 10.1002/ctm2.1525

**Published:** 2024-01-09

**Authors:** Andreas Neueder, Philipp Nitzschner, Ronja Wagner, Julia Hummel, Franziska Hoschek, Maximilian Wagner, Alshaimaa Abdelmoez, Björn von Einem, G. Bernhard Landwehrmeyer, Sarah J. Tabrizi, Michael Orth

**Affiliations:** ^1^ Department of Neurology Ulm University Hospital Ulm Germany; ^2^ Department of Pharmaceutical Organic Chemistry Assiut University Assiut Egypt; ^3^ UCL Huntington's disease Centre UCL Queen Square Institute of Neurology and National Hospital for Neurology and Neurosurgery Queen Square London UK; ^4^ Swiss Huntington Centre Neurozentrum, Siloah AG Gumligen Switzerland; ^5^ University Hospital of Old Age Psychiatry and Psychotherapy Bern University Bern Switzerland

Dear Editor,

In the present study, we demonstrate that examining extracellular vesicles (EVs) can give new insights into pathologic mechanisms in Huntington disease (HD), and might be used as biomarkers. We generated multi‐omics datasets using enriched and purified EVs that were then evaluated with advanced bioinformatics and supervised machine learning to reveal HD‐specific alterations in EV biology. We show that EVs from HD individuals convey specific actionable information in comparison to EVs from healthy people highlighting the biological relevance and potential use of EVs as a biomarker in clinical trials.

The CAG repeat expansion mutation in the huntingtin gene (*HTT*) causing HD is expressed in all tissues. HD is primarily a neurodegenerative, mixed movement disorder, however, non‐neuronal tissues such as liver and skeletal muscle also display various pathological changes.^1^ In line with this, we have recently shown that molecular signatures of inflammation, energy metabolism and vesicle biology are different in peripheral tissues of *HTT* mutation carriers (MTM‐HD study).^2^

EVs are secreted by most, if not all cells. They are composed of lipids, proteins, RNAs and other small molecules and are generally categorised into three subtypes: exosomes, ectosomes and apoptotic bodies.^3^ EVs transfer their cargo by fusing with the recipient cell. Since all EVs are generated with material from the secreting cell, analysis of their composition potentially allows insights into the molecular state of these cells, including in the context of disease.^3^

Following the updated guidelines of the International Society for Extracellular Vesicles (MISEV2018),^4^ we established a robust, fast and highly standardised threefold purification procedure for EVs of all types from human plasma (Figure 1A). Our combined strategy markedly reduced free protein content, especially contaminants such as serum albumin, immunoglobulins or Golgi proteins in the final EV preparation (Figures 1B and S1). The final EV fraction was positive for EV marker proteins LAMP1/CD107a and ICAM1/CD54 by western blotting (Figure S1), and in proteomics data (e.g., ITGA*, ACT*, TUB*, HSP*) demonstrating successful purification of EVs. Transmission electron microscopy of purified, negatively stained EVs showed the expected cup shaped morphology (Figure S2).

**FIGURE 1:**
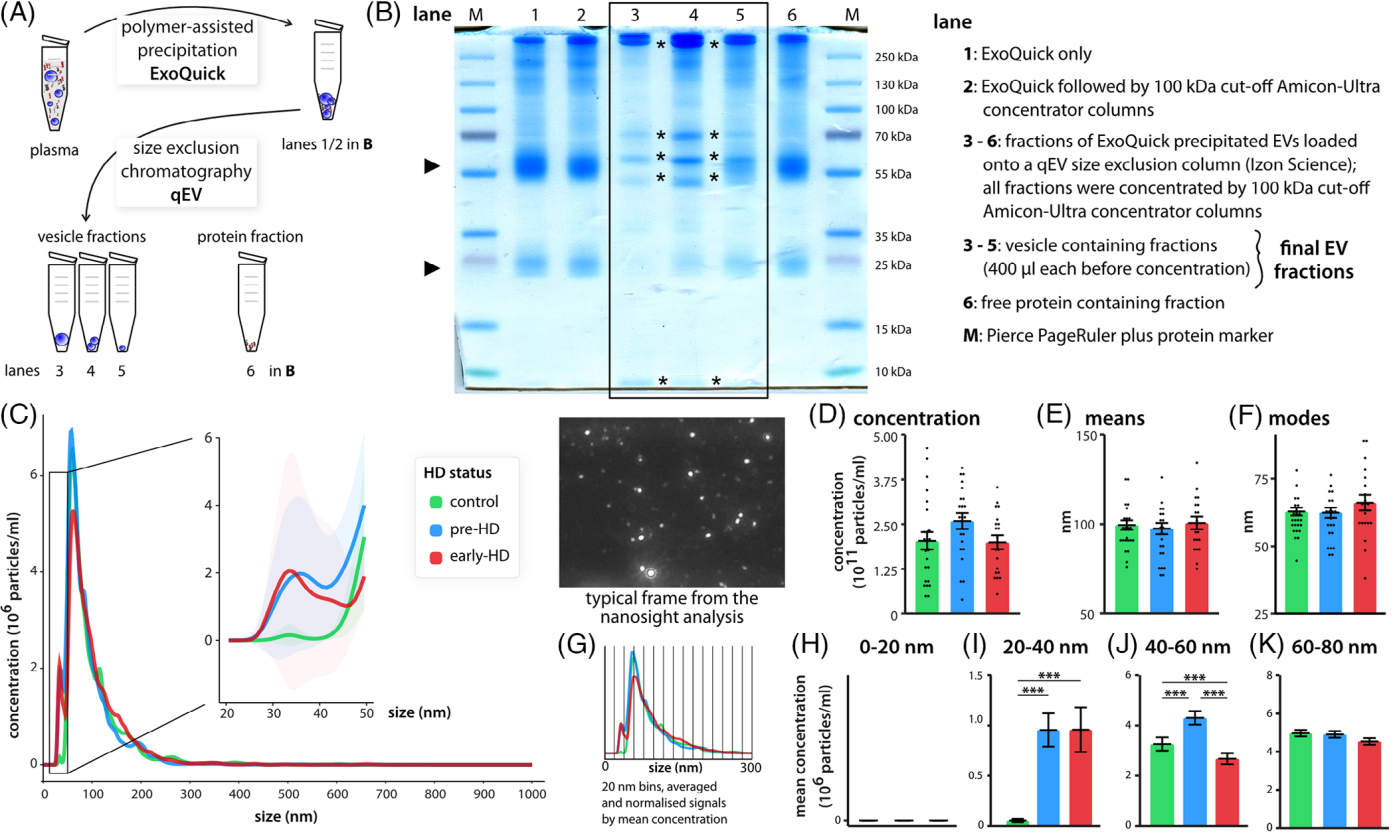
Purification strategy and extracellular vesicle (EV) size distribution differences in *HTT* mutation carriers. (A) Schematic depicting the general purification strategy of EVs from human plasma. (B) Coomassie‐stained gel highlighting the successful isolation of EVs. EV‐specific bands (*) appear in the final EV fractions. (C) Size distribution of EVs from control individuals, *HTT* mutation carriers before symptom onset (pre‐HD) and with early onset (early‐HD) as measured by nanoparticle tracking analysis (nanosight). The traces represent the mean of all samples for each genotype (control, *n* = 24; pre‐HD, *n* = 22; early‐HD, *n* = 20). The inset shows particles with sizes in the range of 20–50 nm. Shading represents the 95% confidence intervals per group. Overall concentration (D), mean (E) and mode (F) diameter were not different between genotypes. Data are mean ± SEM with individual data points shown. One‐way analysis of variance (ANOVA). (G) Strategy to statistically assess size differences in 20 nm size bins between genotypes. (H) We detected no sub 20 nm particles. There were significantly more small particles in the 20−40 nm range for both Huntington disease (HD) groups (I) and in the 40−60 nm range for the pre‐HD group (J). (K) We did not detect any statistically significant differences between genotypes at 60−80 nm and larger (not shown). Data are mean ± SEM. One‐way ANOVA with Tukey post hoc test.

We then used plasma samples from the large MTM‐HD study to purify EVs (control, *n* = 24; pre‐HD, *n* = 22; early‐HD, *n* = 20).^2^ Purification of EVs from 500 µL of plasma was sufficient to isolate enough EVs for all analyses from the same sample including for proteomics and transcriptomics. This avoids the influence of a batch effect due to different purifications. Nanoparticle tracking analysis (Figure 1C) showed that sizes of EVs were consistent with the expected mixture of exosomes and ectosomes. We neither detect larger EVs (apoptotic bodies) in any of the samples nor differences in the overall EV concentration, mean or mode diameter when comparing the three groups (Figure 1). However, we noticed a marked increase in small particles in the two HD groups (Figure 1C, inset), in particular for particles in the size range between 30 and 50 nm (Figure 1I,J). Above 60 nm, we did not detect any statistically significant group differences (Figure 1K).

Next, we generated proteomics and RNAseq datasets from the purified EVs (Figure 2). Using STRING network analysis of the proteomics data, each network exhibited significantly more edges than what would be expected from a random set (Figure 2A–D). Gene ontology analysis using Enrichr^5^ showed a high expression of many dysregulated proteins in liver indicating that liver might be the main source of the observed changes in EV protein content and composition.

**FIGURE 2:**
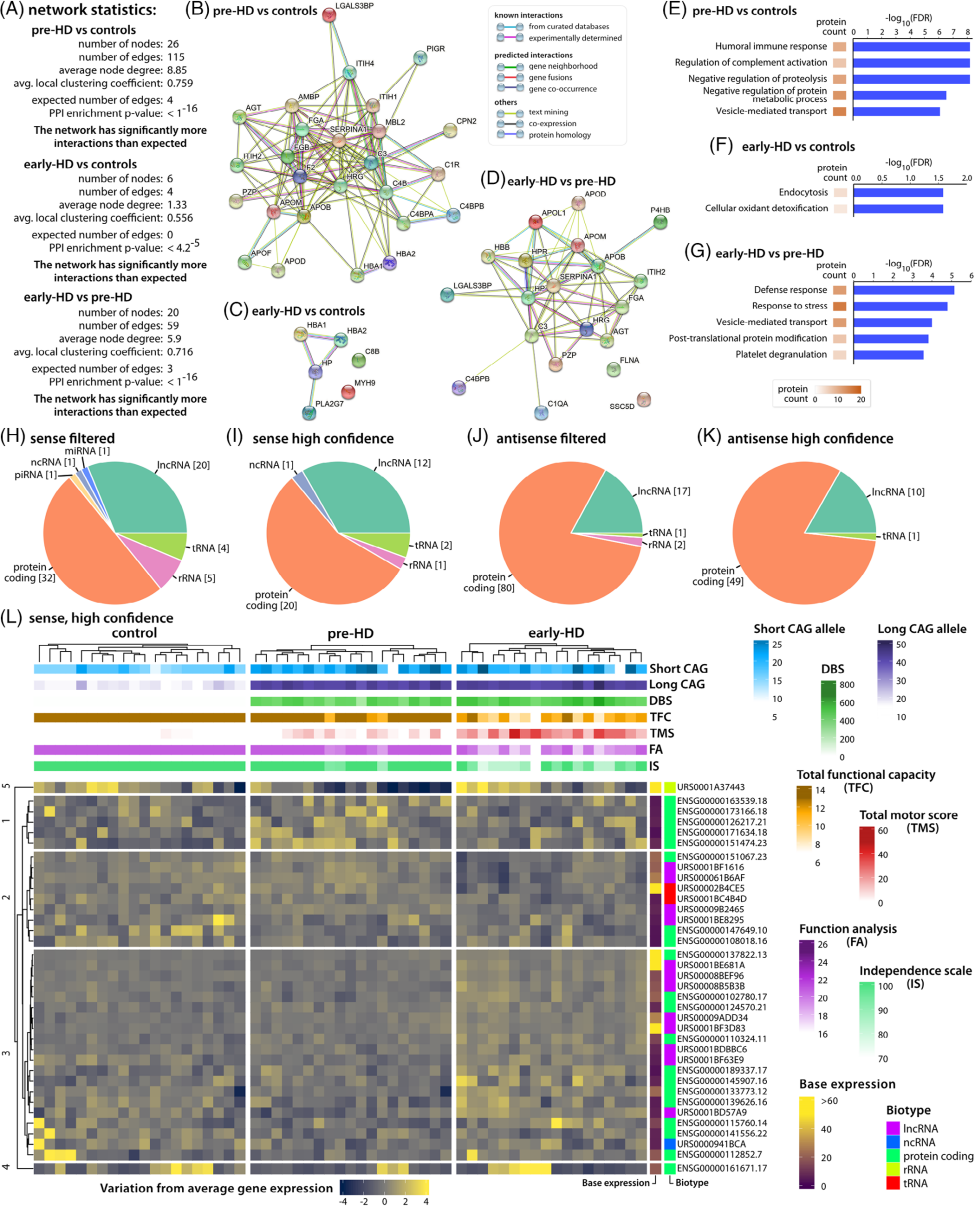
Proteomics and RNAseq analysis of purified extracellular vesicles (EVs). (A) Network statistics from the STRING analysis for all pair‐wise comparisons of the proteomics data. All networks showed significant enrichment over expected number of interactions. (B–D) STRING networks. Interactions between proteins are shown in colour for pre‐HD compared to controls (B), early‐HD compared to controls (C) and early compared to pre‐HD groups (D). (E–G) Ontology enrichment of the pair‐wise comparisons. The first five significantly, non‐redundant enriched gene ontology terms are shown. (H) Biotypes of significantly dysregulated (*p* < .001) sense mapping RNAs for all pair‐wise comparisons in the filtered dataset (expression in approximately 25% or more of the samples/group). (I) Biotypes of significantly dysregulated (*p* < .001) sense mapping RNAs for all pair‐wise comparisons in the high confidence dataset (expression in approximately 60% or more of the samples/group). (J) Biotypes of significantly dysregulated (*p* < .001) antisense mapping RNAs for all pair‐wise comparisons in the filtered dataset (expression in approximately 25% or more of the samples/group). (K) Biotypes of significantly dysregulated (*p* < .001) antisense mapping RNAs for all pair‐wise comparisons in the high confidence dataset (expression in approximately 60% or more of the samples/group). Biotypes were extracted from the GENCODE and RNAcentral gene annotation files. (L) Expression matrix and correlation to clinical parameters of the significantly dysregulated high confidence sense mapping RNAs. K‐mer clustering showed 3 main clusters of expression patterns. Cluster 1 predominantly contained RNAs that were upregulated in pre‐HD samples; cluster 2 predominantly contained RNAs that were downregulated in one or both Huntington disease (HD) groups; cluster 3 predominantly contained RNAs that were upregulated in early‐HD samples.

Our strategy for the analysis of EV‐associated RNAs was designed to identify all types of RNAs, coding and non‐coding (Figures S3 and S4) including *HTT* itself (Figure S5). Dysregulation analysis for any of the pair‐wise comparisons showed several dozen significantly dysregulated transcripts with different biotypes for both, sense and antisense mapping. This was reflected in the high confidence datasets mapping RNAs (Figure 2H–K).

K‐mer clustering showed three main clusters of expression patterns for the high confidence dataset for sense mapping RNAs with differential regulation between groups (Figure 3L). These RNAs included also the *G3BP1* mRNA (greater than twofold upregulated in early‐HD), which was previously implicated in EV biology in HD.^6^ Further analysis of the clusters pointed towards regulation of these genes by REST, a transcriptional repressor involved in expression of cell identity genes and epigenetics,^7^ which we also had identified by transcriptomic changes in adipose tissue from *HTT* mutation carriers.^2^

**FIGURE 3:**
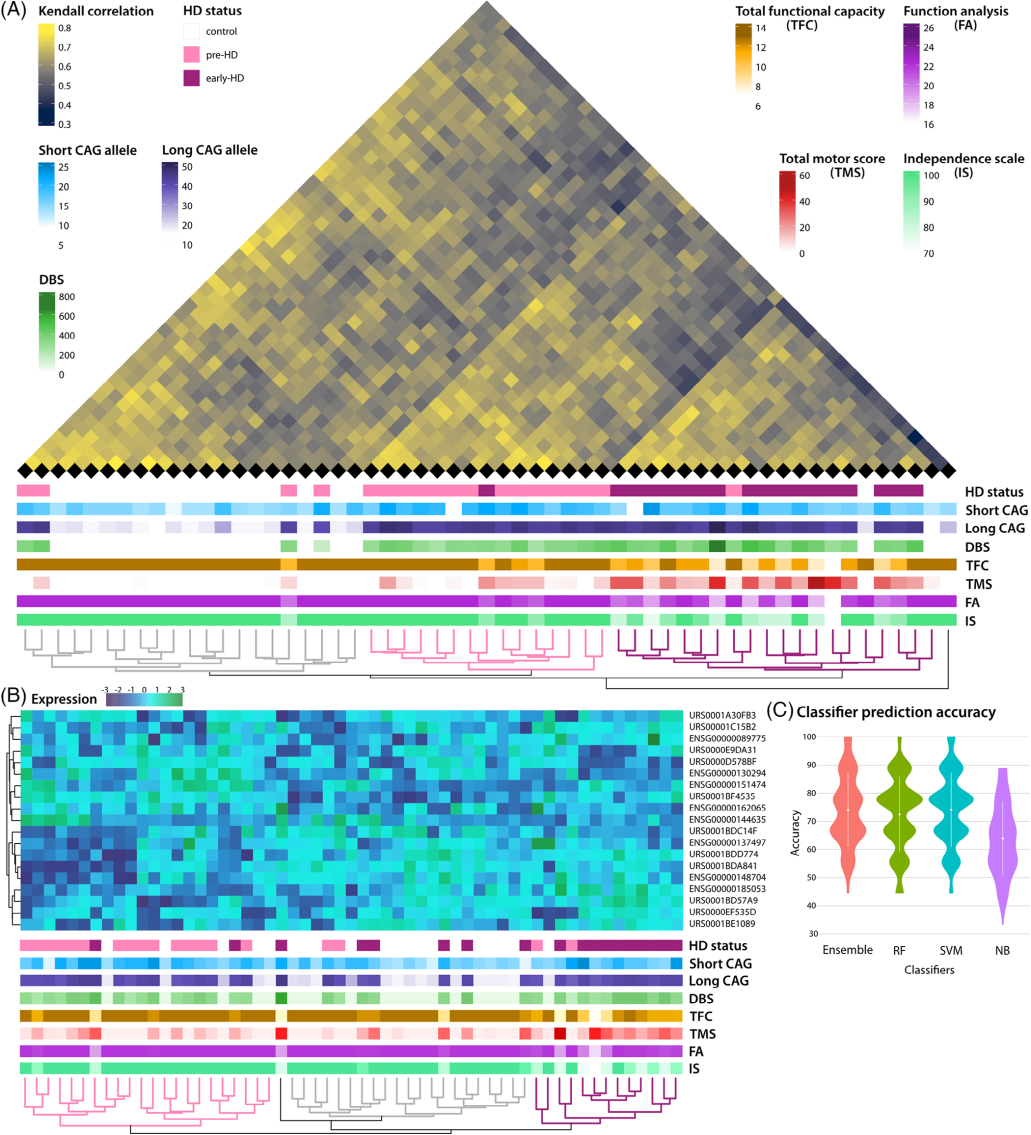
Supervised machine learning based classifier prediction. (A) Unsupervised, rank‐based Kendall clustering of samples based on the filtered dataset (expression in approximately 25% or more of the samples/group) for sense mapping RNAs. Correlation with clinical parameters is shown underneath. Dendrograms are coloured according to the genotype of the majority of assigned samples in each of the three main clusters. (B) Supervised machine learning‐based classifier prediction based on the high confidence dataset filtered (expression in approximately 60% or more of the samples/group) for sense mapping RNAs. The expression matrix (deviation of expression from average expression) for the 19 classifier RNAs is shown with correlation to clinical parameters underneath (colours and scales as in A). Dendrograms are colored according to the genotype of the majority of assigned samples in each of the three main clusters. (C) Violin plots of classifier prediction accuracy for 100 iterations with 90% of the samples as training set and 10% as unknows. Classifier accuracy for the ensemble, as well as random forest (RF), support vector machines (SVM) and naive bayes (NB) classifiers are shown.

We hypothesised that the RNA content of EVs could be used as an HD stage biomarker. To test this hypothesis, we used the expression data from the filtered datasets to generate unsupervised, rank‐based Kendall clustering of the sample relationships (Figures 3A and S6). EV RNAs RNA content was indeed able to accurately distinguish between the groups (Figure 3A). To examine whether a small subset of RNAs, as would be more practical in a clinical setting, would have similar predictive value of HD stage designation, we used a supervised machine learning approach and generated a classifier consisting of only 19 RNAs (Figure 3B) that was able to distinguish the groups with good accuracy (Figure 3C).

In our final set of experiments, we generated proof‐of‐concept data that the purified plasma EVs retained their ability to be taken up by other cells (Figure 4A,B). We showed that the EVs carry actionable information resulting in alterations of the host cell's transcriptome and an EV specific, but genotype independent response. Additionally, we showed a different regulation of genes depending on the genetic origin of the EVs with EVs from early‐HD clearly inducing a distinct response in comparison to EVs from healthy individuals (Figure 4C–E).

**FIGURE 4:**
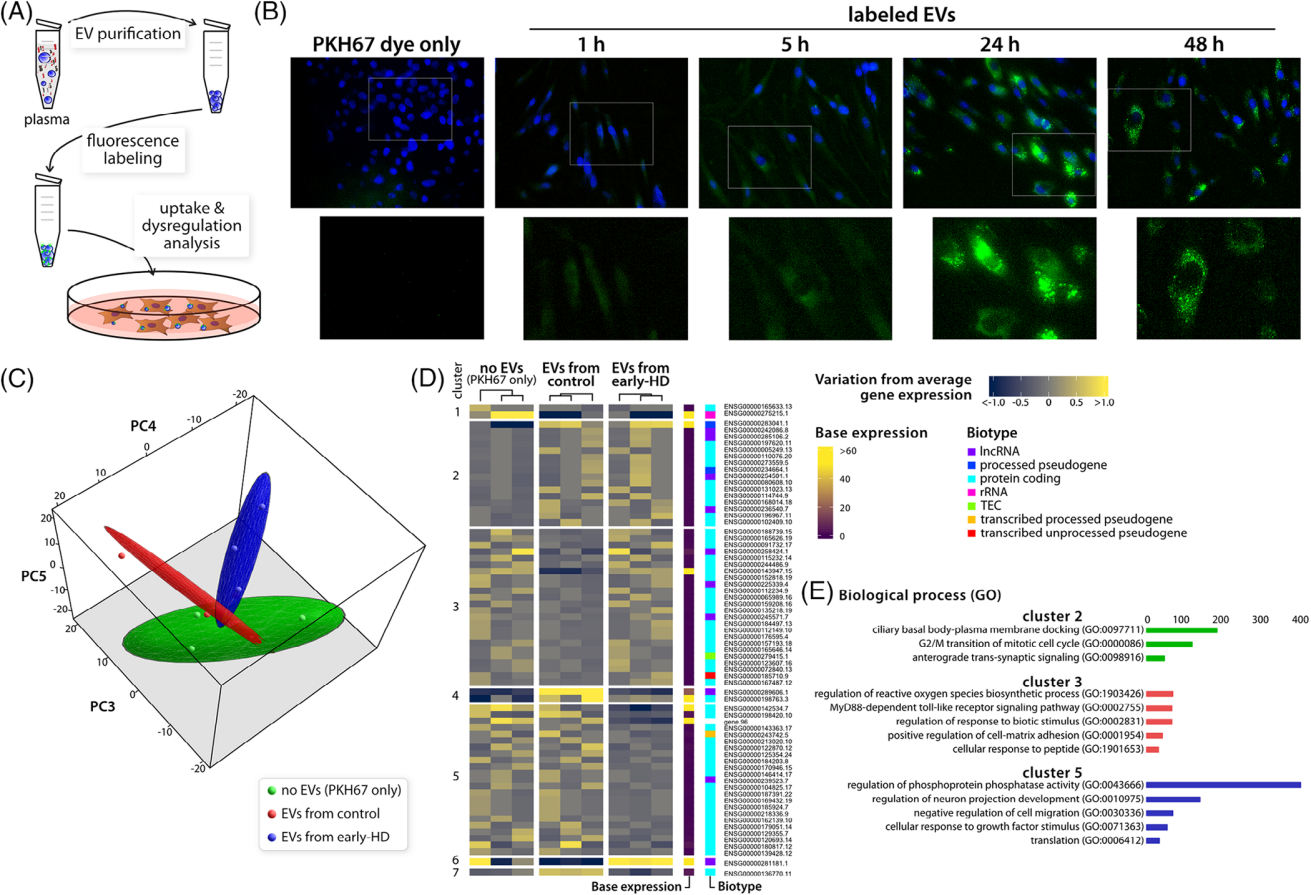
Purified extracellular vesicles (EVs) contain actionable information. (A) Schematic of uptake and dysregulation experiments. (B) Time course of purified, labelled plasma EV uptake in human primary control fibroblasts. Localised high signals were detected after 24 h of EV exposure. (C) 3D principal component (PC) analysis of treated human primary control fibroblasts. Treatment was either no EVs (PKH67 only), or EVs from control or early‐HD samples, respectively. *N* = 3 independent experiments of EV purifications and treatments. PC dimension 3 explained 16.9%, dimension 4 explained 16.2% and dimension 5 explained 15.0% of the observed variance in the dataset. Dimension 4 was significantly associated with EVs from early‐HD individuals compared to control EVs (*p* = .021). Dimension 5 was significantly associated with EVs from controls compared to no EVs (*p* = .021). Spearman's rank correlation test. (D) Heatmap showing the significantly dysregulated genes (*p* < .001) for all three treatment groups. The analysis showed three main clusters of dysregulated genes. Cluster 2: genes upregulated by EV exposure, independent of the genotype of EV genetic origin. Cluster 3: genes downregulated by control EV exposure. Cluster 5: genes downregulated by early‐HD EV exposure. (E) Gene ontology (GO) analysis of identified clusters using Enrichr (GO biological process). Only terms with at least two genes per set were kept. Combined score is the log of Fisher's exact test multiplied by the *z*‐score of the deviation from the expected rank. The larger the combined score, the more significant is the enrichment. Only top five non‐redundant terms with a combined score >5 are shown. HD, Huntington disease.

While some limitations remain, for example, co‐purified lipoproteins, which pose a challenge in EV‐related research and are present to some extent in our EV preparations (independent of genotype), our approach has uncovered previously unidentified HD‐related biological changes: the appearance of small particles in the HD samples, potentially corresponding to exomeres.^8^ A key question concerns the origin of EVs. We deliberately did not enrich subpopulations of EVs in order to retain the maximum information in this first comprehensive analysis. In our proteomics dataset, we detected L1CAM and N1CAM, both of which are potential markers for EVs of neuronal origin. However, expression levels were quite low as none of the label free quantification (LFQ) values for these two proteins passed quality control. We did not detect ATP1A3, which recently was proposed as another neuronal marker for EVs.^9^ In summary, we think that only a very small subset of the EVs we purify is of neuronal origin. In contrast, the ontology analysis points towards liver as the main source of the changes. Liver also exhibits a high degree of somatic CAG repeat instability, a key driver of the events that lead to HD manifestations. The liver therefore might be in a biologically more advanced HD state similar to neuronal populations.^10^

Taken together, our RNA data indicate that peripheral EVs are indeed an attractive source of easily accessible biomarkers since *HTT* sequences and/or potentially *HTT*‐derived small RNAs are associated with EVs. Moreover, the classifier we developed can distinguish *HTT* mutation carriers at a molecular level with very high sensitivity in the prodromal stages of HD before the onset of diagnostic motor signs, that is, in the population most likely to participate in future clinical trials aiming at disease modification. Our data also show that EVs can in principle transfer information from one cell type to another. This information is different for EVs from *HTT* mutation carriers indicating that mutant HTT impacts cell‐to‐cell communication.

Our current data are supportive of a peripheral HD phenotype, ranging from the tissue molecular signatures we have previously reported to plasma EVs. This emphasises the relevance of the expression of *HTT* in non‐neuronal cells, and the effects the mutation causing HD can have on them. It also offers an opportunity to harness such signatures in the form of EVs as biomarkers in clinical trials.

## CONFLICT OF INTEREST STATEMENT

A.N. acted as a consultant for Triplet Therapeutics, Inc. during the time of the study. In the past 2 years, through the offices of UCL Consultants Ltd., a wholly owned subsidiary of University College London. S.J.T. has undertaken consultancy services for Alnylam Pharmaceuticals Inc., Atalanta Pharmaceuticals, F. Hoffmann‐La Roche Ltd., Genentech, Guidepoint, Horama, Locanobio, LoQus23 Therapeutics Ltd., Novartis Pharma, PTC Therapeutics, Sanofi, Spark Therapeutics, Takeda Pharmaceuticals Ltd., Triplet Therapeutics, University College Irvine and Vertex Pharmaceuticals Incorporated. G.B.L. has provided consulting services, advisory board functions, clinical trial services and/or lectures for Acadia Pharmaceuticals, Affiris, Allergan, Alnylam, Amarin, AOP Orphan Pharmaceuticals AG, Bayer Pharma AG, Boehringer‐Ingelheim, CHDI‐Foundation, Deutsche Huntington‐Hilfe, Desitin, Genentech, Genzyme, GlaxoSmithKline, F. Hoffmann‐LaRoche, Ipsen, ISIS Pharma (IONIS), Lilly, Lundbeck, Medesis, Medivation, Medtronic, NeuraMetrix, Neurosearch Inc., Novartis, Pfizer, Prana Biotechnology, Prilenia, PTC Therapeutics, Raptor, Remix Therapeutics, Rhône‐Poulenc Rorer, Roche Pharma AG Deutschland, Sage Therapeutics, Sanofi‐Aventis, Sangamo/Shire, Siena Biotech, Takeda, Temmler Pharma GmbH, Teva, Triplet Therapeutics, Trophos, UniQure and Wave Life Sciences. B.v.E. is a part‐time employee of NatIgGs GmbH. All other authors declare they have no conflicts of interest.

## ETHICS STATEMENT

The local ethics committees at Ulm University and University CollegeLondon approved collection and analysis of the specimens (Ulm: 265‐12; London:12/LO/1565), and written informed consent was obtained from each participant. All experimental methods comply with the Helsinki Declaration.

## Supporting information

Supporting Information

Supporting Information

Supporting Information

Supporting Information

Supporting Information

Supporting Information

Supporting Information

Supporting Information

Supporting Information

Supporting Information

## Data Availability

The datasets supporting the conclusions of this article are included within the article and its additional files. RNAseq raw data are available from GEO accession GSE217159. Proteomics raw data are available from MassIVE accession MSV000090662. Further details about the bioinformatics evaluation, as well as scripts and code are available upon reasonable request from A.N. (andreas.neueder@uni‐ulm.de).
