# Supplementary material for: Huntington disease alters the actionable information in plasma extracellular vesicles
Source: Clin Transl Med. 2024 Jan 9;14(1):e1525. doi: 10.1002/ctm2.1525 (PMC10775183; doi:10.1002/ctm2.1525)
Supplement: Supplementary file 1 — Supporting Information [file CTM2-14-e1525-s008.docx]

Supporting Information

Huntington disease alters the actionable information in plasma extracellular vesicles

Andreas Neueder^1,^*****, Philipp Nitzschner^1^, Ronja Wagner^1^, Julia Hummel^1^, Franziska Hoschek^1^, Maximilian Wagner^1^, Alshaimaa Abdelmoez^1,2^, Björn von Einem^1^, G. Bernhard Landwehrmeyer^1^, Sarah J. Tabrizi^3^ and Michael Orth^1,4,5^

^1^Department of Neurology, Ulm University Hospital, 89081 Ulm, Germany

^2^Department of Pharmaceutical Organic Chemistry, Assiut University, Assiut, Egypt

^3^UCL Huntington's disease Centre, UCL Queen Square Institute of Neurology and National Hospital for Neurology and Neurosurgery, Queen Square, London, WC1N 3BG, United Kingdom

^4^Swiss Huntington Centre, Neurozentrum, Siloah AG, Worbstr. 312, 3073 Gümligen, Switzerland

^5^University Hospital of Old Age Psychiatry and Psychotherapy, Bern University, Bern, Switzerland

***Correspondence**

Andreas Neueder

Department of Neurology, University Hospital Ulm, 89081 Ulm, Germany

P: +49 731 500 63117

E: [andreas.neueder@uni-ulm.de](mailto:andreas.neueder@uni-ulm.de)

ORCID: 0000-0002-2389-5236

# Materials and Methods

## Sample collection

Human plasma samples were collected as previously described for the MTM‑HD study ^1^. Participants of the MTM‑HD study were recruited at the departments of neurology of Ulm University (Germany) and University College London (United Kingdom). At both institutions the study followed a standardized protocol and the personnel at either site received on-site training on all aspects of the standardized protocol to ensure samples were collected and processed in exactly the same manner in Ulm and in London. The CAG-tract length was determined for all participants and all participants were clinically assessed as described for the TrackOn and TRACK-HD studies ^2,3^. Clinical assessment included the United Huntington Disease Rating Scale (UHDRS) motor part to derive the total motor score and the UHDRS total functional capacity scale (TFC) ^4^. The disease burden score (DBS) was calculated from each HD participant's CAG repeat length and age according to the following formula: (CAG-35.5) x age ^5^. *HTT* mutation carriers were categorized as pre‑HD if they had a diagnostic confidence level score of 2 or less on the UHDRS motor scale, or as early‑HD if they were in TFC stages of 1 or 2, indicative of early motor manifest HD. All blood samples were collected following an overnight fast (water was permitted) between 7.30 and 9 am local time (CET or GMT) to account for any influence of circadian rhythm. Blood samples were drawn into BD Vacutainer K_2_EDTA tubes (BD), and plasma was separated by centrifugation at 4° within 30 minutes. Routine blood tests were performed (full blood count, renal, liver, bone and clotting profiles, creatinine kinase and group and save). All samples were then snap frozen in liquid nitrogen and stored at -80˚C.

## EV purification

For purification of EVs, plasma samples were defrosted on ice, centrifuged for 10 mins at 3000 g, 4°C and the supernatant (0.5 ml) transferred to new tube. ExoQuick (System Biosciences) reagent was added (20% of the plasma sample volume, i.e. 100 µl) and samples were incubated on ice for 30 minutes. The samples were centrifuged for 2 mins at >14000 g, 4°C and the supernatant discarded. The pellet was thoroughly resuspended in 150 µl of filtered PBS (0.20 µm, DPBS without Mg2+/Ca2+, Life Technologies). The sample was then transferred to a qEV column (qEVsingle / 70nm, Izon Science). The sample was run into the column by gravity flow. After this filtered PBS was used as separation buffer and fractions were collected. The first 1 ml was discarded; all other fractions contain EVs (next 1 ml) or protein (everything after the first ml). To ensure successful separation, fractions were measured with a nanodrop ND1000 (Peqlab) at 280 nm. To concentrate the diluted EV fraction, we used 100 kDa cut-off spin filters (Amicon Ultra-0.5 Centrifugal Filter Unit, 100 kDa cutoff, Merck) sequentially with 500 µl sample for each spin. Samples were centrifuged for 5 mins at >14000 g, flow-through was discarded. Columns were inverted and final EV samples were eluted by centrifugation for 2 mins at 400 g at room temperature into a protein LoBind tube (Eppendorf). Volume averaged about 80 μl [70 to 90 μl].

## Nanoparticle tracking analysis

After purification, the concentration and size distribution of the EVs were measured using a NanoSight NS300 (Malvern Panalytical). Samples were 1:500 diluted in filtered PBS (0.20 µm, DPBS without Mg2+/Ca2+, Life Technologies) before measurement. Each sample was measured 5 times for 1 minute each in constant flow mode using an attached syringe pump. Data export was through the Nanoparticle tracking analysis v3.40 software. Run data was read into R and screened for outliers per group with a Grubbs test. Summary statistics, as well as binning, were also conducted in R.

## EV marker analysis - Coomassie stain and western blotting

Purified EVs were lysed in HU buffer (200 mM Tris-Cl pH 6.8, 8 M urea, 5 % (w/v) SDS, 1 mM EDTA pH 8.0, 215 mM β-mercaptoethanol) and protein concentration was measured with a nanodrop ND1000 (Peqlab) at 280 nm. Proteins were denatured for 15 minutes at 65°C. Similar amounts of proteins were loaded onto a Mini-PROTEAN TGX precast gel (Bio-Rad) und run under denaturing conditions (50 mM Tris, 50 mM MOPS, 0.1 % (w/v) SDS, 1 mM EDTA, pH 7.7). Gels were either stained with Coomassie (EZBlue, Merck) or transferred onto a nitrocellulose membrane (0.45 µm, Bio-Rad) with transfer buffer (25 mM Tris, 192 mM glycine, pH 8.3) at 450 mA for 45 minutes using the Criterion transfer system (Bio-Rad). The buffer system used for subsequent steps was TBS (50 mM Tris-Cl pH 7.4, 150 mM NaCl) with 0.1 % (w/v) Tween-20 added for washing, or 5% (w/v) skim milk powder for blocking and antibodies incubation, respectively. Antibodies and dilutions were as follows: Anti-GOLGA2 1:1000 (GM130 (D6B1), #12480, Cell Signaling), anti-ICAM1 1:500 (CD54/ICAM-1, #4915, Cell Signaling), anti-albumin 1:5000 (MAB1455, R&D Systems), anti-LAMP1 1:1000 (LAMP-1/CD107a, MAB4800, R&D Systems). All primary antibody incubations were overnight at 4°C in blocking buffer.

## Proteomics

Following protein precipitation, EV samples were reconstituted in 100 mM ammonium hydrogen carbonate. After reduction and alkylation, proteins were digested overnight using a trypsin to protein ratio of 1:50. The resulting peptides were lyophilized, reconstituted in 15 µl 5% (v/v) trifluoroacetic acid and analyzed via HPLC-MS employing an LTQ Orbitrap Elite system as described previously ^6^. Database search was performed using MaxQuant Ver. 1.6.3.4 ^7^ employing the built-in Andromeda search engine ^8^. For peptide identification, MS/MS spectra were correlated with the UniProt human reference proteome set (www.uniprot.org) (version 26^th^ Nov. 2018, 73940 protein entries). Carbamidomethylated cysteine was considered as a fixed modification. Methylation, oxidation and protein N-terminal acetylation were considered as variable modifications. For label free quantification (LFQ) values were generated by Andromeda.

## Proteomics evaluation

LFQ intensities were used for quantification. Out of the 951 identified proteins for the 66 samples (24 controls, 22 pre-HD and 20 early-HD), all proteins with 65 or 66 missing values were discarded. Contaminants (e.g. keratins, hornerin) were removed. The remaining 294 proteins were clustered according to genotype (control, pre-HD, early-HD) and only proteins that were identified in at least 5 samples (approximately 25%) per group were analyzed further (158 proteins). LFQ signals were normalized and transformed with a variance stabilizing normalization (vsn) v3.60.0 ^9^. Cluster analysis based on the Euclidean distance with a z of -3.5 identified three outlier samples (IDs: MTMHD 03, 04 and 35), leaving final sample sizes of 22 controls, 20 pre-HD and 20 early-HD samples. Following this, we consecutively removed batch effects with limma v3.42.2 ^10^: Sex, site of sampling, BMI, age. To compute protein dysregulation, we used DEqMS v1.10.0 ^11^. All proteins with a *p*-value < 0.05 were used as input for STRING analysis v11.5 ^12^.

## RNA extraction from purified EVs

RNAs were extracted from freshly purified EVs without any freeze/thaw steps in between. To this end, 350 μl of lysis buffer from the SeraMir Exosome RNA Amplification kit (System Biosciences) was added to the purified EVs, vortexed for 15 sec at maximum speed, incubated for 5 mins at room temperature and 200 μl of 100% ethanol (RNA grade) added. The sample was vortexed for 10 sec at maximum speed and the precipitating solution was transferred to an ExoRNA spin column. Samples were bound and washed twice (each 400 µl of wash buffer) with centrifugation steps of 1 min at > 15.000 g. Flow-throughs were discarded. Columns were dried 2 min at > 15.000 g and placed into a new DNA low-bind tube (Eppendorf). 23 μl of RNase free water was directly applied onto the middle of the column without touching it and incubated for 1 min at room temperature. RNAs were eluted by centrifugation for 2 min at < 400 g, followed by centrifugation for 1 min at > 15.000 g. RNA was aliquoted, frozen in liquid nitrogen and stored at -80°C. RNA quality assessment and quantification were carried out using a Agilent 2100 Bioanalyzer with RNA 6000 pico chips (Agilent Technologies).

## Transcriptomics (RNAseq)

RNAs were extracted as described above. The sequencing library was prepared using the SMARTer smRNA-Seq Kit (Takara Bio, Kusatsu, Japan) with 100 pg total RNA as input according to the manufacturer's protocol. In short, adenylation of the 3' ends enabled priming of the reverse transcription reaction using an oligo(dT) primer containing the Read 2 adapter sequence for sequencing. Subsequently, cDNA synthesis was performed, making use of the template-switching technology and adding the Read 1 sequencing adapter. Libraries were then amplified in a PCR with 21 cycles using barcoded primers. Eight differently barcoded forward (i5 index) primers and twelve different reverse (i7 index) primers provided in the kit were used in 66 different combinations to enable multiplexing of all 66 samples on one flowcell. After the PCR, samples were cleaned up using the column-based NucleoSpin Gel and PCR Clean-Up kit (Macherey-Nagel, Düren, Germany). The double two-sided size selection described in the user manual was omitted to also retain larger molecules. The molarity of the libraries was assessed using the High Sensitivity NGS assay of the FragmentAnalyzer (Agilent, Santa Clara, USA) for average fragment size, and fluorometric quantitation to measure library concentration with the dsDNA High Sensitivity assay on a Qubit 3.0 (both Thermo Fisher Scientific, Waltham, MA, USA). The libraries were pooled in an equimolar fashion, denatured according to the manufacturer's instructions and diluted to 270 pM. Sequencing was performed on a NovaSeq 6000 (Illumina, San Diego, CA, USA) as 150 bp paired-end reads with an average sequencing depth of 11 Mio clusters (i.e. read-pairs) per sample.

## Transcriptomics evaluation

After demultiplexing, samples were trimmed using cutadapt v3.2 ^13^ as follows: Read 1: Remove everything 3' of A_10_. Read 2: Remove everything 3' of AGATCGGAAGAGCGTCGTGTAGGGAAAGAGTGT; on the reverse complementary reads remove everything 3' of A_10_. For final read pairs: In paired end mode keep only reads with a minimum length of 15 base pairs; remove 3 nucleotides from the 5' ends of the reads. FastQ files were quality controlled with FastQC v0.11.8 ^14^. A merged gene annotation was prepared for use with salmon v1.5.2 ^15^. To this end, the transcript fasta sequences from the GENCODE human release 38 (https://www.gencodegenes.org/human) and the human specific transcript fasta sequences from RNAcentral v19 release (https://rnacentral.org/) were merged. Together with primary assembly genome fasta file (GENCODE) and its decoys, these were used to generate a salmon index. For assessment of optimal kmer length, salmon v1.4.0 and GENCODE human release 37 transcripts were used to generate the indices. To quantify sense transcripts, salmon was run with the -l ISF option, for antisense transcripts with the -l ISR option. Common parameters were --validateMappings --softclip --softclipOverhangs --recoverOrphans --incompatPrior 0.0 --seqBias --gcBias --posBias --numBootstraps 100. Quantification files were imported using tximport v1.20.0 ^16^ and collapsed to gene level with the non-coding RNA transcripts of the RNAcentral database considered as 'genes'. Variance stabilizing transformed (VST) ^17^ counts from an intercept matrix in DESeq2 v1.32.0 ^18^ were used to correct for batch effects (site of sampling, BMI, sequencing batch, sex and age) in limma 3.48.3, and a Euclidean distance with a z of -3 was used to screen for outliers. Two outliers were identified and removed (1 control and 1 early‑HD) resulting in final group sizes of: Controls n = 20, pre‑HD n = 19 and early‑HD n = 18. Dysregulated genes (sense and antisense) were computed with DESeq2 with the aforementioned batch effects included in the design formula and ashr as the shrinking estimator ^19^. To generate heatmaps and for classifier prediction, the same procedure as for outlier screening was followed, except excluding the two outlier samples. Heatmaps were generated with ComplexHeatmap v2.12.0 ^20^. We removed genes with expression in less than approximately 25% of the samples per group (n ≥ 5 per genotype required for inclusion) to generate the 'filtered' dataset. The 'high confidence' dataset required reads in approximately 60% of the samples per group (n ≥ 12 for controls and n ≥ 11 for pre‑HD and early‑HD).

## Supervised machine learning based classifier prediction

To predict a classifier subset of RNAs, we used the 'high confidence' datasets of variance stabilized transformed, batch corrected counts as input for DaMiRseq v2.4.3 ^21^. Feature selection (DaMiR.FSelect) was run with minimum Spearman correlation between class and PCs of 0.6 and a threshold setting of 1.5, with 1 iteration. This discarded 8,473 genes for classification and 395 genes remained. Reduction and sorting were run with default parameters resulting in 332 remaining genes. Selection of best predictors (DaMiR.FBest) with a z-score > 1.5 resulted in 19 remaining genes. For assessment of classifier prediction accuracy, we used the DaMiR.EnsembleLearning function with a split of the dataset in 90% for known (training) and 10% unknown samples for 100 iterations.

## Human primary fibroblast cell culture, EV labeling and uptake assay

Human primary fibroblasts were maintained in DMEM with high glucose and L-glutamine (BE12-741F, Lonza) supplemented with 15% FBS (FBS Supreme, South America, PAN-Biotech), 1 mM pyruvate (Sodium pyruvate, Gibco), 1x non‑essential amino acids (MEM-NEAA 100x, Gibco) and 1% penicillin/streptomycin (ThermoFisher). Cells were grown at 37°C, 5% CO_2_ in a humid incubator until around 90% confluency before passaging. All cell lines were routinely controlled for mycoplasma, fungal and bacterial contamination.

EVs were purified as described above from selected individuals with distinct transcriptomic changes. The final EV samples were adjusted to 500 µl with filtered PBS (0.20 µm, DPBS without Mg2+/Ca2+, Life Technologies). 2 µl of PKH67 dye (Merck) in 500 µl diluent C were added and the solution was thoroughly mixed and incubated for 5 minutes at room temperature. The solution was consecutively (2x 500 µl) centrifuged through a concentrator column (Amicon Ultra-0.5 Centrifugal Filter Unit, 100 kDa cutoff, Merck), 5 minutes at >14000 g, room temperature. Samples were eluted (2 minutes at 400 g, room temperature), again adjusted to 500 µl with filtered PBS and re-applied to the same concentrator column. After centrifugation for 5 minutes at >14000 g, room temperature, the final, labelled EVs were eluted by centrifugation for 2 minutes at 400 g, room temperature. The amount of labelled EVs from initially 500 µl of plasma was used to treat a T75 flask with approximately 80-90% confluent human primary fibroblasts. For time course analysis of EV uptake, cells were grown on poly-L-lysine coated coverslips and the labelled EV amount was adjusted accordingly to the growth area.

## Nanopore sequencing

RNA from human primary fibroblasts was extracted using the RNeasy Plus Mini Kit (Qiagen) according to manufacturer's instructions. RNA was eluted in water and quantified with the Qubit RNA BR Assay (Thermo Scientific). RNA was precipitated overnight in 0.3 M NaCl in 70% EtOH at -20°C. RNA was pelleted with centrifugation for 30 min at >14000 g at 4°C. The RNA pellet was resuspended in 16 µl water. 2.25 µg of RNA was used as input for library preparation with the direct cDNA Sequencing kit (SQK‑DCS109, Oxford Nanopore Technologies) according to the manufacturer's protocol. cDNA synthesis including strand switch was performed with Maxima H Minus reverse transcriptase (ThermoFisher). The RNA was degraded (RNase Cocktail Enzyme Mix, ThermoFisher) and a second strand was synthesized (LongAmp Taq, New England Biolabs). Double stranded cDNA was then used for end-prep (NEBNext Ultra II End repair / dA-tailing Module, New England Biolabs) and barcodes were ligated (NEB Blunt/TA Ligase Master Mix, New England Biolabs). All wash steps were conducted using Agencourt AMPure XP beads (Beckman Coulter). The samples were mixed in equimolar amounts and divided into three equivalents for re-loading of the flowcells. The multiplexed libraries were stored at -20°C until further use. For sequencing, one multiplexed library was thawed, AM II adapter ligation was performed (NEBNext Quick Ligation Module, New England Biolabs) and the library was bound to loading beads and loaded for sequencing on a nanopore Mk1C. After 24 and 48 hours, flowcells were washed and the same library was re-loaded.

## Nanopore sequencing evaluation

Nanopore reads were base-called and demultiplexed using guppy v6.1.3 (Oxford Nanopore Technologies). Samples were quality controlled with pycoQC v2.5.0.3 ^22^. Alignment was computed with minimap2 v2.22 ^23^ against GENCODE human release 40 (https://www.gencodegenes.org/human). Gene counts were summarized using bambu v2.2.0 ^24^ and dysregulation was computed using DESeq2 v1.36.0 ^18^ with passage of the cells as a covariate. 3D PCA plots were generated using pcaExplorer v2.22.0 ^25^ and rgl v0.109.2 ^26^. Heatmaps were generated using ComplexHeatmap v2.12.0 ^20^. Input for the PCA analysis and heatmaps were VST transformed counts from an DESeq2 v1.36.0 ^18^ intercept design, subsequently batch corrected with limma v3.52.2 ^10^ for passage number.

# Ethics approval and consent to participate

The local ethics committees at Ulm University and University College London approved collection and analysis of the specimens (Ulm: 265-12; London: 12/LO/1565), and written informed consent was obtained from each participant. All experimental methods comply with the Helsinki Declaration.

# Authors' contributions

A.N. and M.O. conceptualized the project. A.N. developed the detailed study design. A.N., P.N., R.W., J.H., F.H., M.W., A.A. and B.v.E. generated and analyzed data. A.N. and M.O. provided supervision and mentorship. S.J.T and M.O. provided resources. A.N. drafted the manuscript and prepared the figures. M.O. contributed to critical revision of the manuscript. Each author contributed important intellectual content during manuscript drafting or revision and accepts accountability for the overall work by ensuring that questions pertaining to the accuracy or integrity of any portion of the work are appropriately investigated and resolved. All authors approved the final version of the report. The corresponding author (A.N.) attests that all the listed authors meet the authorship criteria and that no others meeting the criteria have been omitted.

# Supplementary Figures and Figure legends


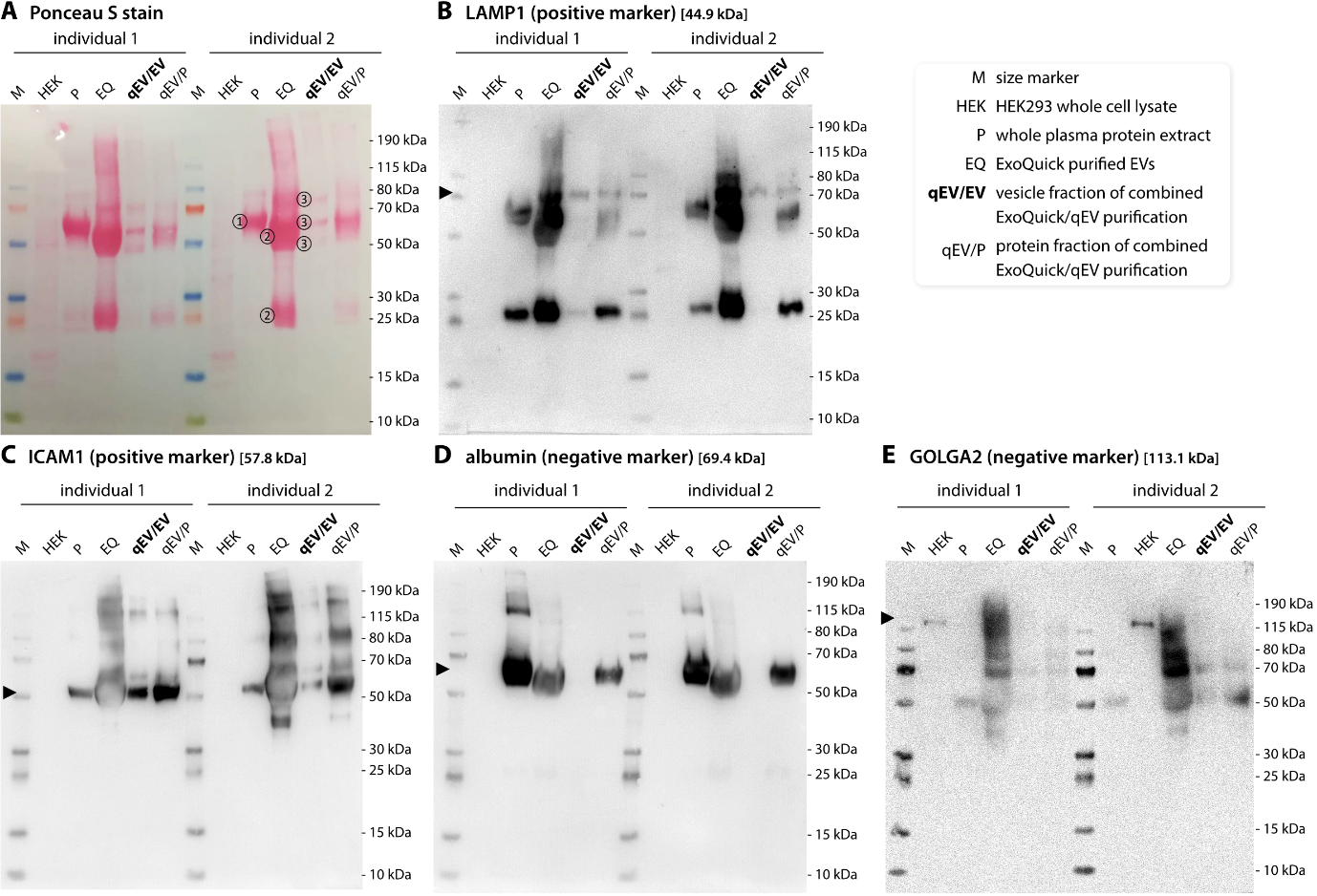


## Figure S1. EV protein analysis by western blotting confirms the presence of EV markers.

EVs from plasma of two healthy individuals were purified and analyzed by western blotting with the indicated antibodies. 10 µg of total protein per lane were loaded. The final EV fractions (qEV/EV) are highlighted in bold. (A) Exemplary Ponceau S stain of a membrane after transfer. In plasma (P) the most abundant protein is albumin ①. In the ExoQuick EV fraction (EQ) the most abundant proteins are immunoglobulins (② heavy and light chain). The final EV fraction (qEV/EV) exhibits EV specific bands ③. (B) LAMP1/CD107a is a positive marker for EVs. It is heavily glycosylated and runs at a higher apparent weight of about 80 kDa. (C) ICAM1/CD54 is a positive marker for EVs. (D) Albumin is a negative marker for EVs. The band in the EQ fraction could be a cross-reaction of the anti-albumin primary or anti-mouse secondary antibodies with the very abundant immunoglobulin heavy chains (see A). (E) GOLGA2/GM130 is a negative marker for EVs. The protein could only be detected in a HEK293 whole cell lysate (HEK). The black triangles (►) point at the apparent molecular weight of the analyzed proteins.


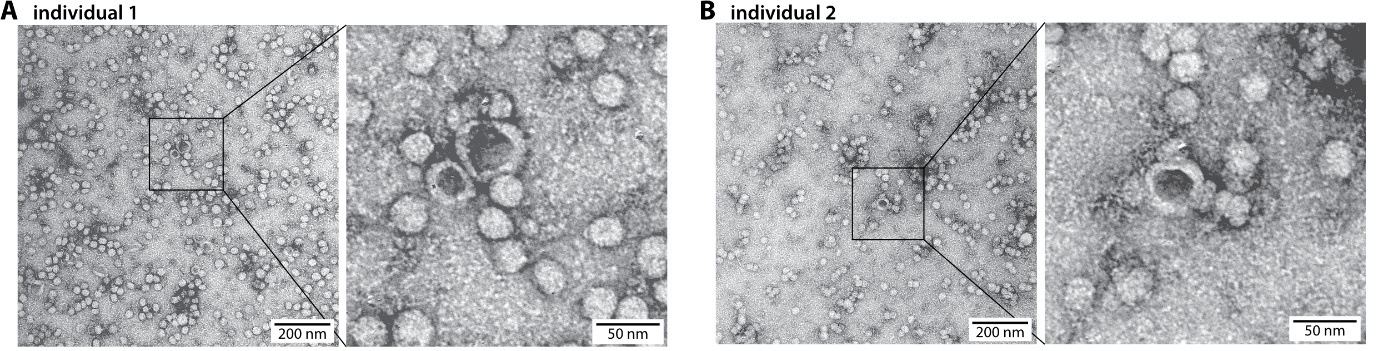


## Figure S2. Transmission electron microscopy images of purified EVs show the expected morphology.

(A and B) EVs were imaged by negative staining (osmium tetroxide) on a JEM 2100 (JEOL) electron microscope. EVs were purified from plasma of two healthy individuals (A and B, respectively). The right panel is a higher magnification zoom of an area in the left panel. EVs display the typical cup shaped morphology as usually observed in transmission electron microscopy.


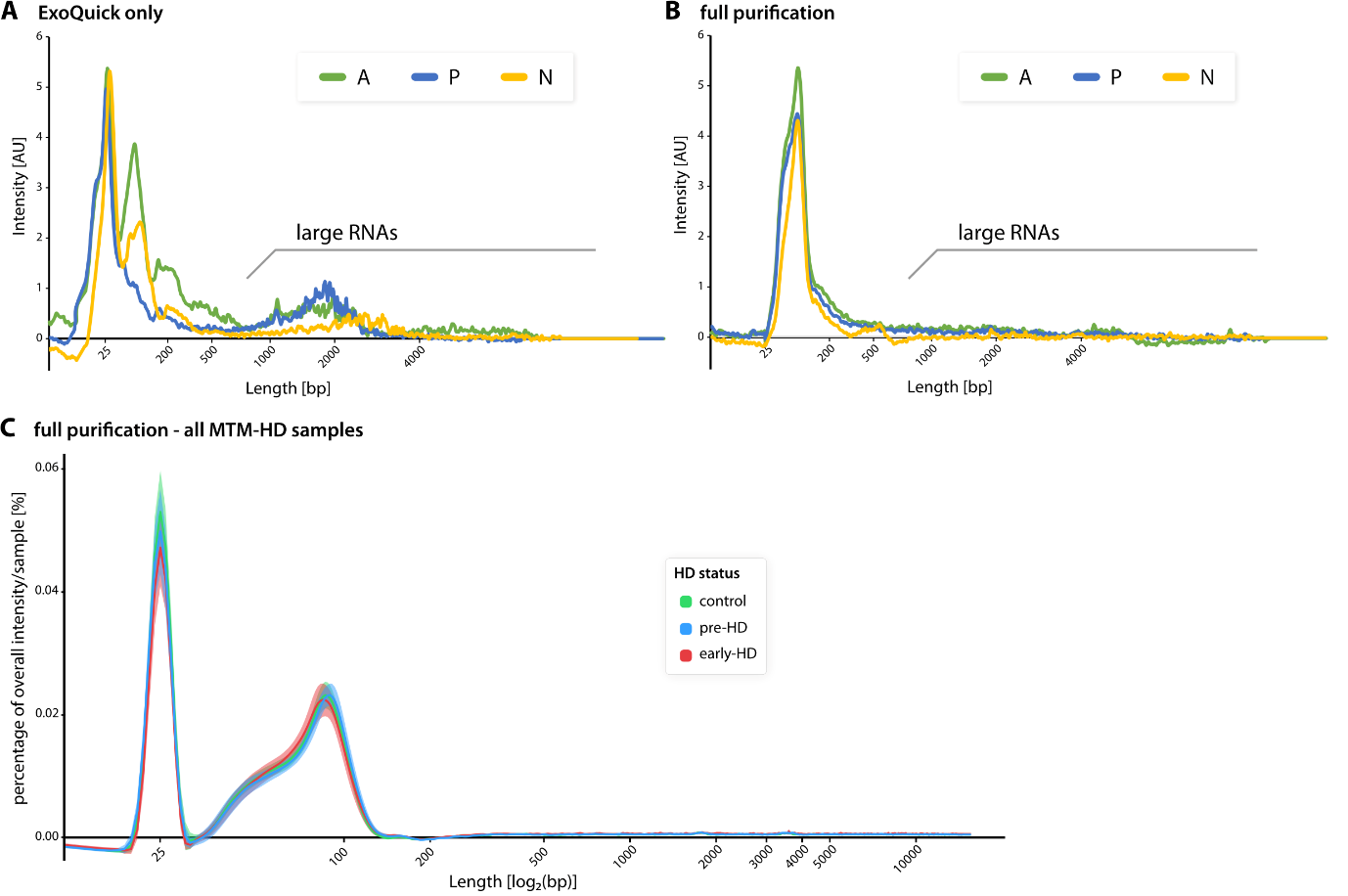


## Figure S3. RNA profile analysis by bioanalyzer measurements

(A and B) Bioanalyzer RNA traces of two EV purification strategies from the same three healthy individuals (A, P and N). (A) RNAs extracted from EVs that were purified by precipitation (ExoQuick) only. These EV RNAs correspond to lane 1 in Fig. 1B. (B) RNAs extracted from EVs that were purified with our final protocol (precipitation followed by size exclusion chromatography and ultrafiltration). These EV RNAs correspond to lanes 3-5 in Fig. 1B. Note the distinct lack of large RNA species. (C) Bioanalyzer RNA traces by groups with 95% confidence intervals for each group as shades. The lines represent the average of all samples per group (control n = 21; pre‑HD n= 19; early‑HD n = 19). These RNAs were sequenced in the transcriptomics analysis.


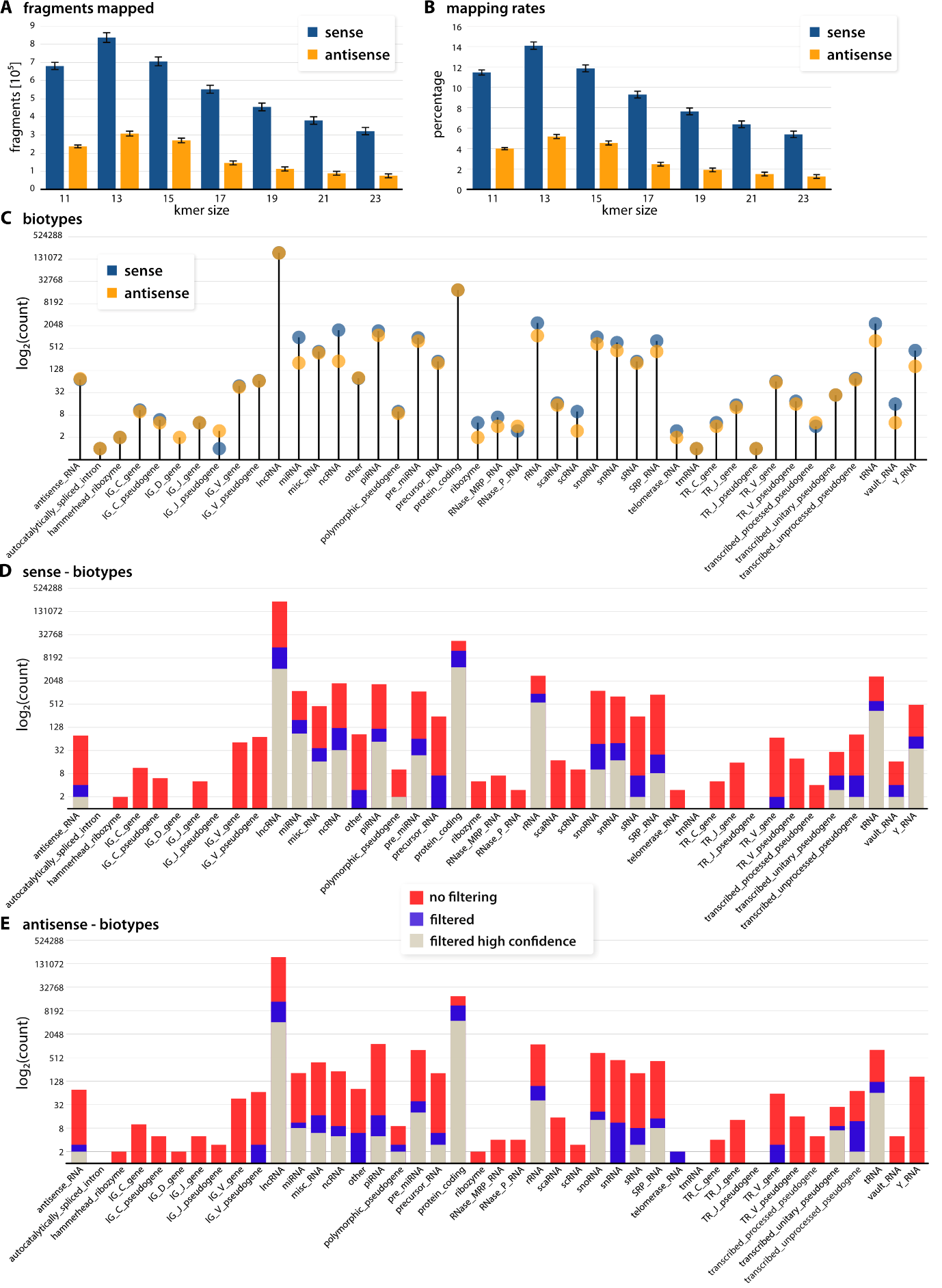


## Figure S4. RNAseq mapping rates and identified biotypes.

(A) Number of fragments mapped for different k-mer lengths used to generate the salmon indices (see also Materials and Methods section). (B) Mapping rates in percent of total number of read pairs after trimming for different k-mer lengths used to generate the salmon indices. (C) Associated biotypes of all identified sense and antisense mapping RNAs. Biotypes were extracted from the GENCODE and RNAcentral gene annotation files. (D and E) Comparison of biotypes without filtering, in the filtered (expression in approximately 25% or more of the samples/group) and in the high confidence (expression in approximately 60% or more of the samples/group) dataset for sense (D) and antisense (E) mapping RNAs.


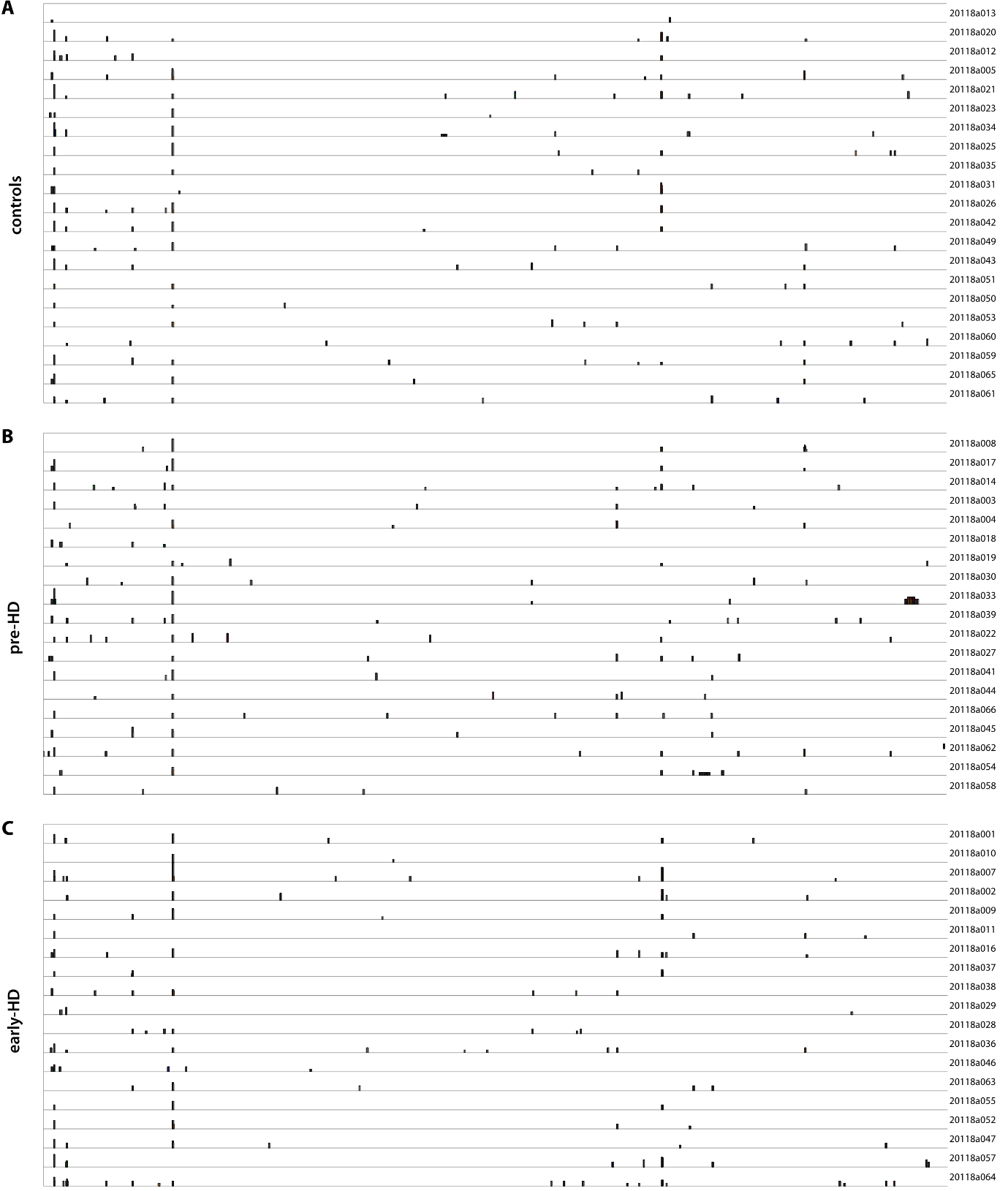


## Figure S5. RNAseq reads mapping to *HTT*-201 (ENST00000355072.11).

Alignment of RNAseq reads to the main, full-length *HTT* transcript as generated by salmon during its mapping stage of the quantification for control (A), pre‑HD (B) and early‑HD samples (C). Y-axis is 0 to 64 reads for each sample in logarithmic scale for better visibility. Read coverage is before normalization for library size by DEseq2. Sample names on the right correspond to column names in supplementary data file 4.


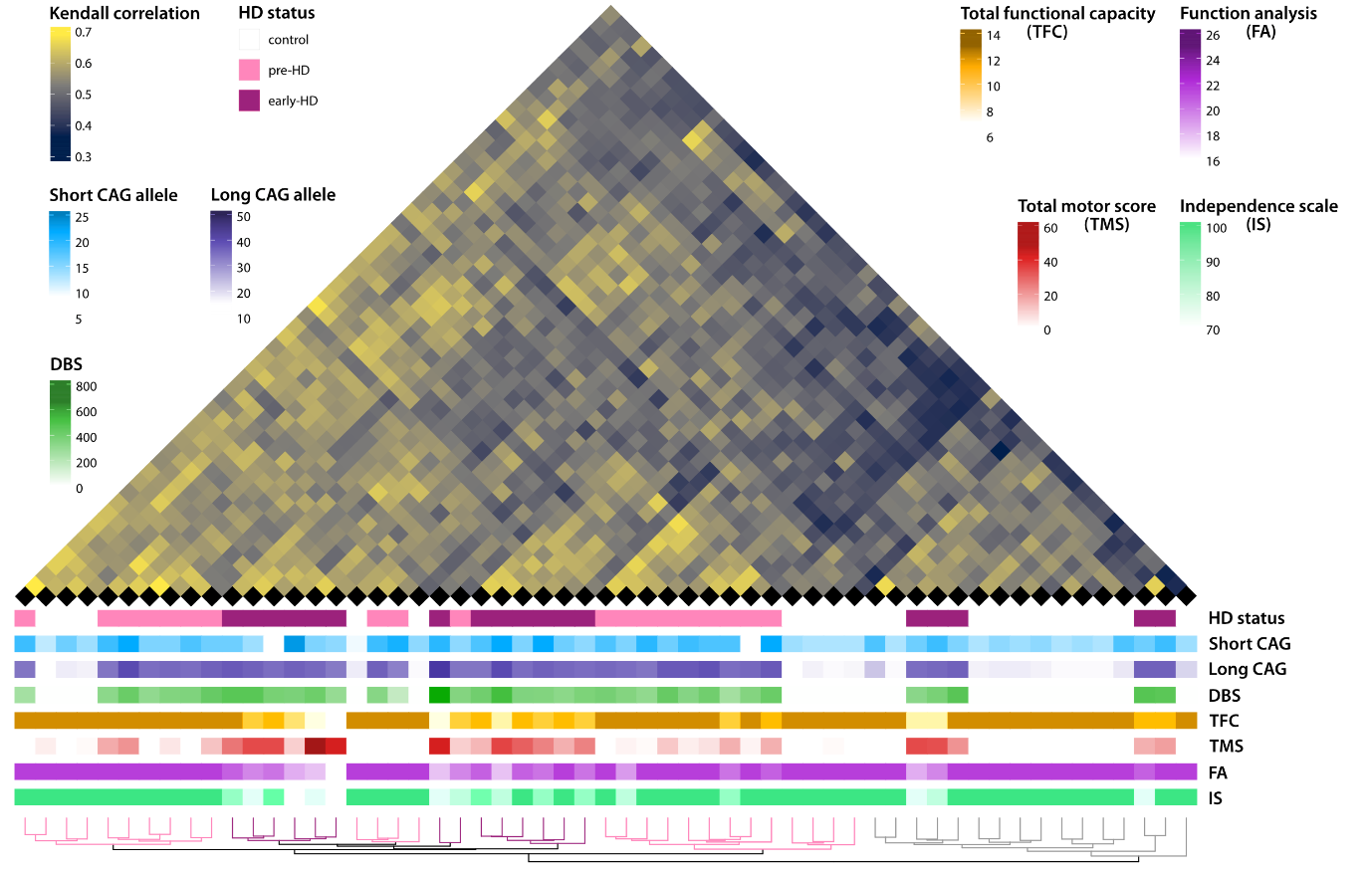


## Figure S6. Sample relationships based on antisense mapping RNAs.

Unsupervised, rank based Kendall clustering of samples based on the filtered dataset (expression in approximately 25% or more of the samples/group) for antisense mapping RNAs. Correlation with clinical parameters is shown underneath. Dendrograms are colored according to the genotype of the majority of assigned samples in each of the clusters.

# Supplementary data files

Supplementary data file 1 - Clinical data associated with the samples used in this study.

Supplementary data file 2 - Proteomics raw data; LFQs per sample

Supplementary data file 3 - DEqMS proteomics evaluation results

Supplementary data file 4 - RNAseq raw data; normalized counts per sample, outliers removed; sense (ISF) mapping RNAs

Supplementary data file 5 - RNAseq raw data; normalized counts per sample, outliers removed; antisense (ISR) mapping RNAs

Supplementary data file 6 - DESeq2 RNAseq evaluation results for sense (ISF) mapping RNAs

Supplementary data file 7 - DESeq2 RNAseq evaluation results for antisense (ISR) mapping RNAs

Supplementary data file 8 - Metadata and unnormalized, non-corrected raw counts per gene for the EV uptake and dysregulation experiments

Supplementary data file 9 - DESeq2 evaluation results for the EV uptake and dysregulation experiments

# Supplementary references

1. Neueder A, Kojer K, Hering T, et al. Abnormal molecular signatures of inflammation, energy metabolism, and vesicle biology in human Huntington disease peripheral tissues. *Genome Biol*. Sep 7 2022;23(1):189. doi:10.1186/s13059-022-02752-5

2. Orth M, Gregory S, Scahill RI, et al. Natural variation in sensory-motor white matter organization influences manifestations of Huntington's disease. *Hum Brain Mapp*. Dec 2016;37(12):4615-4628. doi:10.1002/hbm.23332

3. Tabrizi SJ, Langbehn DR, Leavitt BR, et al. Biological and clinical manifestations of Huntington's disease in the longitudinal TRACK-HD study: cross-sectional analysis of baseline data. *Lancet Neurol*. Sep 2009;8(9):791-801. doi:10.1016/S1474-4422(09)70170-X

4. Huntington Study Group. Unified Huntington's Disease Rating Scale: reliability and consistency. Huntington Study Group. *Movement disorders : official journal of the Movement Disorder Society*. Mar 1996;11(2):136-42. doi:10.1002/mds.870110204

5. Penney JB, Jr., Vonsattel JP, MacDonald ME, Gusella JF, Myers RH. CAG repeat number governs the development rate of pathology in Huntington's disease. *Annals of neurology*. May 1997;41(5):689-92. doi:10.1002/ana.410410521

6. Arnold F, Gout J, Wiese H, et al. RINT1 Regulates SUMOylation and the DNA Damage Response to Preserve Cellular Homeostasis in Pancreatic Cancer. *Cancer research*. Apr 1 2021;81(7):1758-1774. doi:10.1158/0008-5472.CAN-20-2633

7. Cox J, Mann M. MaxQuant enables high peptide identification rates, individualized p.p.b.-range mass accuracies and proteome-wide protein quantification. *Nature biotechnology*. Dec 2008;26(12):1367-72. doi:10.1038/nbt.1511

8. Cox J, Neuhauser N, Michalski A, Scheltema RA, Olsen JV, Mann M. Andromeda: a peptide search engine integrated into the MaxQuant environment. *Journal of proteome research*. Apr 1 2011;10(4):1794-805. doi:10.1021/pr101065j

9. Huber W, von Heydebreck A, Sultmann H, Poustka A, Vingron M. Variance stabilization applied to microarray data calibration and to the quantification of differential expression. *Bioinformatics*. 2002;18 Suppl 1:S96-104. doi:10.1093/bioinformatics/18.suppl_1.s96

10. Ritchie ME, Phipson B, Wu D, et al. limma powers differential expression analyses for RNA-sequencing and microarray studies. *Nucleic Acids Res*. Apr 20 2015;43(7):e47. doi:10.1093/nar/gkv007

11. Zhu Y, Orre LM, Zhou Tran Y, et al. DEqMS: A Method for Accurate Variance Estimation in Differential Protein Expression Analysis. *Molecular & cellular proteomics : MCP*. Jun 2020;19(6):1047-1057. doi:10.1074/mcp.TIR119.001646

12. Szklarczyk D, Gable AL, Nastou KC, et al. The STRING database in 2021: customizable protein-protein networks, and functional characterization of user-uploaded gene/measurement sets. *Nucleic Acids Res*. Jan 8 2021;49(D1):D605-D612. doi:10.1093/nar/gkaa1074

13. Martin M. Cutadapt removes adapter sequences from high-throughput sequencing reads. next generation sequencing; small RNA; microRNA; adapter removal. *EMBnetjournal*. 2011-05-02 2011;17(1):3. doi:10.14806/ej.17.1.200

14. Andrews S. FastQC: A Quality Control tool for High Throughput Sequence Data. [*http://wwwbioinformaticsbabrahamacuk/projects/fastqc*](http://wwwbioinformaticsbabrahamacuk/projects/fastqc). 2010;doi:citeulike-article-id:11583827

15. Patro R, Duggal G, Love MI, Irizarry RA, Kingsford C. Salmon provides fast and bias-aware quantification of transcript expression. *Nature methods*. Apr 2017;14(4):417-419. doi:10.1038/nmeth.4197

16. Soneson C, Love MI, Robinson MD. Differential analyses for RNA-seq: transcript-level estimates improve gene-level inferences. *F1000Research*. 2015;4:1521. doi:10.12688/f1000research.7563.2

17. Anders S, Huber W. Differential expression analysis for sequence count data. *Genome Biol*. 2010;11(10):R106. doi:10.1186/gb-2010-11-10-r106

18. Love MI, Huber W, Anders S. Moderated estimation of fold change and dispersion for RNA-seq data with DESeq2. *Genome Biol*. 2014;15(12):550. doi:10.1186/s13059-014-0550-8

19. Stephens M. False discovery rates: a new deal. *Biostatistics*. Apr 1 2017;18(2):275-294. doi:10.1093/biostatistics/kxw041

20. Gu Z, Eils R, Schlesner M. Complex heatmaps reveal patterns and correlations in multidimensional genomic data. *Bioinformatics*. Sep 15 2016;32(18):2847-9. doi:10.1093/bioinformatics/btw313

21. Chiesa M, Colombo GI, Piacentini L. DaMiRseq-an R/Bioconductor package for data mining of RNA-Seq data: normalization, feature selection and classification. *Bioinformatics*. Apr 15 2018;34(8):1416-1418. doi:10.1093/bioinformatics/btx795

22. Leger A, Leonardi T. pycoQC, interactive quality control for Oxford Nanopore Sequencing. *Journal of Open Source Software*. 2019;4(34):1236. doi:10.21105/joss.01236

23. Li H. New strategies to improve minimap2 alignment accuracy. *Bioinformatics*. Oct 8 2021;doi:10.1093/bioinformatics/btab705

24. Ying C, Göke J, Sim A, et al. GoekeLab/bambu: bambu v2.0.0-Bioconductor release. *Zenodo*. 2021;doi:10.5281/zenodo.5641944

25. Marini F, Binder H. pcaExplorer: an R/Bioconductor package for interacting with RNA-seq principal components. *BMC Bioinformatics*. Jun 13 2019;20(1):331. doi:10.1186/s12859-019-2879-1

26. Adler D, Nenadí O, Zucchini W. Rgl: A r-library for 3d visualization with opengl. 01/01 2003;
